# Defining cut-off values for disease activity states and improvement scores for patient-reported outcomes: the example of the Rheumatoid Arthritis Impact of Disease (RAID)

**DOI:** 10.1186/ar3859

**Published:** 2012-05-30

**Authors:** Maxime Dougados, Yves Brault, Isabelle Logeart, Désirée van der Heijde, Laure Gossec, Tore Kvien

**Affiliations:** 1 Rheumatology B Department, Paris-Descartes University, Assistance Publique Hôpitaux de Paris, Cochin Hospital, 27 rue du faubourg Saint-Jacques, Paris 14, Paris, 75014, France; 2 Pfizer Ltd France, 23 avenue du Dr Lannelongue, Paris 14, Paris, 75668, France; 3 Rheumatology Department, Leiden University Medical Center, Albinusdreef 2, Leiden, 2333, ZA, Zuid-Holland, Netherlands; 4 Rheumatology Department, Diakonhjemmet Hospital, Diakonveien 12, Oslo, 0370, Norway

## Abstract

**Introduction:**

The Rheumatoid Arthritis Impact of Disease (RAID) is a patient-reported outcome measure evaluating the impact of rheumatoid arthritis (RA) on patient quality of life. It comprises 7 domains that are evaluated as continuous variables from 0 (best) to 10 (worst). The objective was to define and identify cut-off values for disease activity states as well as improvement scores in order to present results at the individual level (for example, patient in acceptable state, improved patient).

**Methods:**

Patients with definite active RA requiring anti-tumour necrosis factor (anti-TNF) therapy were seen at screening, baseline and after 4 and 12 weeks of etanercept therapy. Answers to "Gold standard" questions on improvement (MCII: Minimum Clinically Important Improvement) and an acceptable status (PASS: Patient Acceptable Symptom State) were collected as well as the RAID score and Disease Activity Score 28- erythrocyte sedimentation rate (DAS28-ESR). Cut-offs were defined by different techniques including empirical, measurement error and gold standard anchors. The external validity of these cut-offs was evaluated using the positive likelihood ratio (LR) based on the patient's perspective (for example, patient's global) and on low disease activity status (such as DAS28-ESR).

**Results:**

Ninety-seven (97) of the 108 recruited patients (age: 54 ± 13 years old, female gender: 75%, rheumatoid factor positive: 81%, disease duration: 8 ± 7 years, CRP: 18 ± 30 mg/l, DAS28-ESR: 5.4 ± 0.8) completed the 12 weeks of the study. The different techniques suggested thresholds ranging from 0.2 to 3 (absolute change) and from 6 to 50% (relative change) for defining MCII and thresholds from less than 1 to less than 4.2 for defining PASS. The evaluation of external validity (LR+) showed the highest LR+ was obtained with thresholds of 3 for absolute change; 50% for relative change and less than 2 for an acceptable status.

**Conclusions:**

This study showed that thresholds defined for continuous variables are closely related to the methodological technique, justifying a systematic evaluation of their validity. Our results suggested that a change of at least 3 points (absolute) or 50% (relative) in the RAID score should be used to define a MCII and that a maximal value of 2 defines an acceptable status.

**Trial Registration:**

Clinicaltrial.gov: NCT004768053

## Introduction

During the last decade and in particular since the participation of patients in different Outcome Measures in Rheumatology (OMERACT) activities [1-3] there has been growing interest in the assessment of rheumatoid arthritis (RA) from the patient's perspective. Apart from patient-reported outcomes traditionally evaluated during the current standard assessment of RA, namely patient assessment of pain, functional disability and/or patient global assessment [4,5], other health domains are also important for the patient such as fatigue, wellbeing and sleep pattern [6-8]. Under the umbrella of the European League Against Rheumatism (EULAR), a patient-reported composite index, the Rheumatoid Arthritis Impact of Disease (RAID) score has been proposed and validated [9-12]

This composite index includes seven domains (pain, function, fatigue, physical and psychological wellbeing, sleep disturbance and coping). Each domain is evaluated using a single question answered by a 0 to 10 numerical rating scale. Each domain also has a specific weight assigned by a patient survey. The RAID score is a continuous variable ranging from 0 (best) to 10 (worst)(Table 1).

**Table 1:** Rheumatoid Arthritis Impact of Disease questionnaire*

**1. Pain**												
Circle the number that best describes the pain you felt due to your rheumatoid arthritis during the last week:												
None	0	1	2	3	4	5	6	7	8	9	10	Extreme
**2. Functional disability assessment**												
Circle the number that best describes the difficulty you had in doing daily physical activities due to your rheumatoid arthritis during the last week:												
No difficulty	0	1	2	3	4	5	6	7	8	9	10	Extreme difficulty
**3. Fatigue**												
Circle the number that best describes how much fatigue you felt due to your rheumatoid arthritis during the last week:												
No fatigue	0	1	2	3	4	5	6	7	8	9	10	Totally exhausted
**4. Sleep**												
Circle the number that best describes the difficulty the sleep difficulties (*i.e.*, resting at night) you felt due to your rheumatoid arthritis during the last week:												
No difficulty	0	1	2	3	4	5	6	7	8	9	10	Extreme difficulty
**5. Physical well-being**												
Considering your arthritis overall, how would you rate your level of physical wellbeing during the past week? Circle the number that best describe your level of physical well-being:												
Very good	0	1	2	3	4	5	6	7	8	9	10	Very bad
**6. Emotional well-being**												
Considering your arthritis overall, how would you rate your level of emotional well being during the past week? Circle the number that best describes your level of emotional well-being:												
Very good	0	1	2	3	4	5	6	7	8	9	10	Very bad
**7. Coping**												
Considering your arthritis overall, how well did you cope (manage, deal, make do) with your disease during the last week:												
Very well	0	1	2	3	4	5	6	7	8	9	10	Very poorly

*adapted from Gossec L et al [30]

In general, the results of clinical studies and trials are reported at group level, for example by the mean change from baseline and this makes determination of the relevance of the results for an individual patient challenging. In order to make interpretation easier, data may be presented at individual level by considering the proportion of patients with an improvement above a threshold of an important change from baseline. Moreover, apart from the concept of improvement ('feeling better), the concept of status ('feeling good') has become increasingly important [13]. In order to assess these individual outcomes, continuous outcome measures (absolute value or change in RAID score) for each patient must be converted into a dichotomous variable (that is, change from baseline above a clinically relevant cutoff defining an important improvement from the patient's perspective, or absolute value below a clinically relevant cutoff defining an acceptable or good condition from the patient's perspective). These cutoffs have been called Minimal Clinically Important Improvement (MCII) for improvement and Patient Acceptable Symptom State (PASS) for status [14-17].

Three types of technique have been proposed to determine thresholds. According to the first simple empirical method, an absolute change of at least 1 or 2 points on a 0 to 10 scale [18,19] or a relative change of at least 20, 30 or 50% [20,21] have been proposed as thresholds for several patient-reported outcomes in rheumatic disorders. The second technique considers a change to be relevant when it exceeds the measurement error [22]. The third technique uses a gold standard anchor (usually the patient's global assessment) to determine the threshold from the best ratio between sensitivity and specificity using the receiver operating characteristic (ROC) curve [23], correct classification probabilities [24] or the 75^th ^percentile [14,15].

To our knowledge, despite the recognition of the validity of the RAID questionnaire, no formal threshold has been proposed to present results at the individual level. Furthermore, although several techniques have been used to establish threshold values as detailed above, no comparison has been performed between these techniques.

We were therefore prompted to conduct a study in order to define and evaluate the validity of cutoffs for the RAID score using the different techniques described above.

## Materials and methods

### Study design

This study was a multi-center, open-label, single-arm trial with a screening visit, baseline visit (assuming patient disease activity was stable across the two visits) and visits after 4 and 12 weeks of etanercept therapy (clinicaltrials.gov allocated number NCT 00768053). For each patient, written informed consent was obtained according to the Declaration of Helsinki. The study was approved by the Institutional Review Board of Cochin Hospital, Paris, France.

### Inclusion criteria

To be eligible for the study, patients had to have definite RA fulfilling the 1987 criteria of the American College of Rheumatology [21]. The disease had to be active according to the following definition: Disease Activity Score 28-erythrocyte sedimentation rate (DAS28-ESR) > 3.2 and at least one of the following: ≥ 4 swollen joints or C-reactive protein (CRP) ≥ 10 mg/l or ESR ≥ 28 mm/1^st ^H, and the patient had to be eligible for tumor-necrosis factor (TNF) blocker therapy as recommended by the French Society of Rheumatology [25].

### Collected data

Patients' age, gender and disease characteristics (duration, anti-citrullinated protein antibody (ACPA) status) were collected at screening. The DAS28 [26], modified health assessment questionnaire (mHAQ) [27] and RAID questionnaire were collected at screening, baseline and after 4 and 12 weeks of etanercept therapy. In addition, after 4 and 12 weeks of etanercept therapy, the patients assessed their condition by answering the following dichotomous question: 'If you were to remain during the next few months as you were during the last 48 hours, would this be acceptable to you: yes - no?' [15].

At weeks 4 and 12, patients assessed their change from baseline by answering the following questions: 'Think about all the ways your rheumatoid arthritis has affected you during the last 48 hours. Compared to when you started the study, how have you been during the last 48 hours? a) Improved, b) No change, c) Worse. If you answered "improved" to the previous question, how important is this improvement for you? a) Very important, b) Moderately important, c) Slightly important, d) Not important at all.'

### Statistical analyses

Statistical analysis was conducted in two steps. We first determined thresholds that may be used to define responders (that is, improved patients) and patients in an acceptable condition (that is, good/acceptable status). The improvement threshold was determined by different techniques:

1. An empirical technique based on proposals in the rheumatology scientific literature (for example, an absolute change of at least 1 and 2 in the 0 to 10 RAID score; a relative change of at least 20, 30 and 50% versus baseline) [18-20].

2. A technique based on the reliability of the RAID score considering that a relevant change at the individual level should be at least superior to the measurement error of the technique. For this purpose, the data collected at screening and baseline (interval during which the disease activity was considered stable) were used to assess the relative reliability by calculating the intra-class coefficient of correlation (ICC) with its 95% confidence interval (CI), the absolute reliability based on Bland and Altman plots, presenting the 95% limits of agreement on a graph [22], and the proposed threshold as the smallest detectable change defined as 1.96 × SD of the changes/√2 [28].

3. The third technique was an anchored method based on the patient's perspective. The external anchor was the general question on patient perception of change in comparison to baseline. The threshold RAID score for improvement was determined in three different ways. Firstly the RAID-MCII threshold was determined as the 75^th ^percentile of the distribution of changes in RAID score for patients perceiving a slight or moderate improvement [14]. For this purpose, we considered as a potential threshold the score for which 75% of the patients in the targeted category had a value below this score. The second analysis used the correct classification probabilities [24]. For this purpose, we calculated the sensitivity (percentage of patients with a measured change in RAID score below the threshold for patients considering their condition to be at least slightly or moderately improved) and specificity (percentage of patients with a change in RAID score above the threshold for patients considering their condition to be at least slightly or moderately improved). This was done for a range of possible cutoffs and this was plotted on a graph. The choice of proposed cutoff for this analysis was based on maximal sensitivity and specificity using the graphic representation of correct classification probabilities. The third analysis used the nonparametric ROC curves [23]. The optimal cutoff was determined by minimizing the number of misclassified patients. Such evaluations were performed for the absolute and relative changes after 4 and 12 weeks of etanercept therapy.

Several similar techniques were also used to determine thresholds describing patients with an acceptable status/condition according to the RAID score:

1. Empirical method with thresholds ≤ 1, ≤ 2 and ≤ 3 according to previous proposals in the rheumatology scientific literature.

2. The anchored method based on the patient's perspective using, as an external gold standard, the general question about patients' perceptions of their condition during the 48 hours before the visit. The RAID threshold for acceptable status was determined using the three different analyses described for the improvement (for example, the 75^th ^percentile, the correct classification properties and the ROC curve technique), with the patients considering their condition as acceptable used as the gold standard. All analyses were performed after 4 and 12 weeks of etanercept therapy.

We then evaluated the validity of all the proposed thresholds. Two external anchors were chosen for this purpose. The first was the gold standard from the patient's perspective: for each proposed MCII threshold, we calculated the percentage of patients with a change above the threshold and who considered their condition to be at least slightly improved, among all patients who considered their condition to be at least slightly improved (for example, sensitivity) and also the percentage of patients with a change below the threshold and who considered their condition to be either worse, unchanged or only slightly improved, among all patients who considered their condition to be worse, unchanged or only slightly improved (for example, specificity). The positive likelihood ratio (LR) was then calculated. A LR greater than one indicates an increased probability that the targeted disorder is present. In our study, the use of LR was transposed to express performance of the RAID threshold in reflecting patients' perspectives. Higher values are indicative of better-performing thresholds. All analyses were conducted at 4 and 12 weeks after initiation of etanercept therapy.

A similar analysis was conducted to evaluate the validity of the proposed thresholds of the RAID score to define an acceptable status. Here, the gold standard anchor was based on the patient's perspective by analyzing the patients considering (or not) their situation during the last 48 hours as acceptable. The second external anchor used to evaluate the validity of the proposed thresholds was based on the DAS28-ESR. This composite index is considered to be relatively physician-oriented as it comprises one laboratory measure (ESR) and information collected at physical examination (number of swollen and tender joints) as well as a patient-reported outcome (patient's global assessment). Similar analyses (that is, evaluation of positive LR for the different proposed thresholds evaluating the concept of improvement and status) were conducted as described above.

## Results

### Patients and study course

Of the 120 patients screened, 108 entered the study and received at least one etanercept injection. During the 12 weeks of the trial, one patient was lost to follow-up and ten withdrew because of side effects. The main characteristics of the 108 recruited patients were as follows: age (mean ± SD), 54 ± 13 years; 75% female; 61% ACPA-positive; disease duration, 8 ± 7 years; CRP, 18 ± 30 mg/l, DAS28-ESR, 5.4 ± 0.8.

### Determination of thresholds

#### Relevant improvement threshold

**Figure 1:**
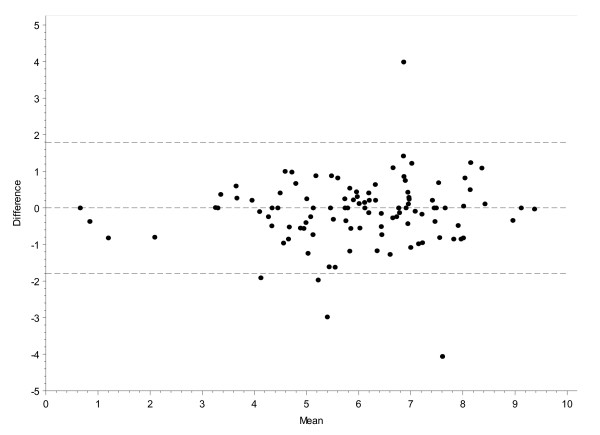
**Reliability of the rheumatoid arthritis impact of disease (RAID) score shown by Bland & Altman graphic representation**. *Mean of RAID score values between screening and baseline; **difference in RAID score between screening and baseline. The data lines represent the 95% confidence interval resulting in a smallest detectable change of 1.3 (for example, smallest detectable difference = 1.8).

The reliability of the RAID score between screening and baseline was very high (ICC = 0.85, 95% CI 0.79 to 0.90). The Bland and Altman graphic representation is illustrated in Figure 1. Using this technique, the smallest detectable difference (SDD) and the smallest detectable change (SDC) in the RAID score were 1.8 and 1.3 respectively.

**Figure 2:**
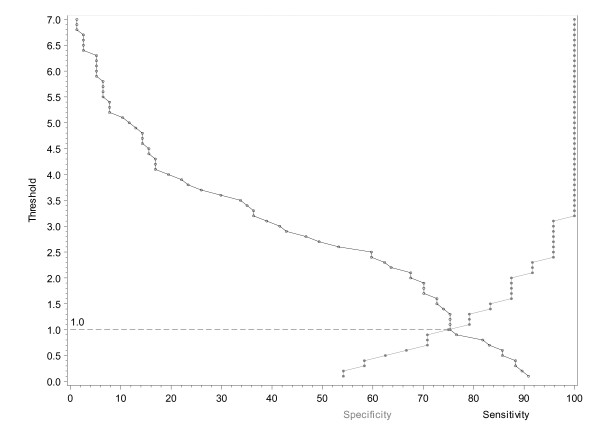
**Correct classification probability curve showing absolute change in rheumatoid arthritis impact of disease (RAID) score at week 4**.

A graphic representation of correct classification probabilities was obtained, based on patient's opinion for the absolute changes in RAID score after 4 weeks of etanercept therapy (Figure 2). The sensitivity and specificity for clinically relevant change was obtained for each measured difference in RAID score (0.1 per 0.1). This made it possible to obtain the best RAID threshold with maximal true positive and minimal false negative results, which was 1.0. A similar analysis was performed after 12 weeks of etanercept therapy and also for the relative change after 4 and 12 weeks of etanercept therapy, resulting in potential thresholds of 2.5, 25% and 42% respectively (Table 2).

**Table 2:** Elaboration and evaluation of the external validity of the different potential thresholds defining a relevant improvement in the rheumatoid arthritis impact of disease (RAID) score

	ELABORATION	Time of evaluation during the study^c^	EVALUATION					
Proposed threshold^a^	Methodological technique^b^		Patient's perspective^d^			Physician's perspective^e^		
			Se	Spe	LR+ (95% CI)	Se	Spe	LR+ (95% CI)
ABSOLUTE CHANGE								
a. ≥ 0.2	75^th ^percentile at week 4	Week 4	89.6	54.2	2.0 (1.3; 3.0)	100.0	29.6	1.4 (1.2; 1.7)
a. ≥ 0.2	75^th ^percentile at week 4	Week 12	96.1	40.9	1.6 (1.1; 2.3)	93.3	17.6	1.1 (1.0; 1.3)
b. ≥ 1	Empirical/Correct classification at week 4	Week 4	75.3	75.0	3.0 (1.5; 6.1)	89.3	47.9	1.7 (1.3; 2.2)
b. ≥ 1	Empirical/Correct classification at week 4	Week 12	90.8	54.5	2.0 (1.3; 3.2)	88.9	27.5	1.2 (1.0; 1.5)
c. ≥ 1.3	SDC/75^th ^percentile at week 12	Week 4	75.3	79.3	3.6 (1.6; 8.0)	85.7	47.9	1.6 (1.3; 2.2)
c. ≥ 1.3	SDC/75^th ^percentile at week 12	Week 12	88.2	59.1	2.2 (1.3; 3.6)	84.4	29.4	1.2 (1.0; 1.5)
d. ≥ 1.6	ROC at week 4	Week 4	72.7	87.5	5.8 (2.0; 16.9)	85.7	53.5	1.8 (1;4; 2.5)
d. ≥ 1.6	ROC at week 4	Week 12	84.2	63.6	2.3 (1.3; 4.1)	82.2	33.3	1.2 (1.0; 1.6)
e. ≥ 2	Empirical/Correct classification at week 4	Week 4	67.5	87.5	5.4 (1.9; 15.7)	78.6	56.3	1.8 (1.3; 2.5)
e. ≥ 2	Empirical/Correct classification at week 4	Week 12	78.9	63.6	2.2 (1.2; 3.8)	77.8	37.3	1.2 (1.0; 1.6)
f. ≥ 2.5	Correct classification at week 12	Week 4	59.7	95.8	14.3 (2.1; 98.5)	71.4	64.8	2.0 (1.4; 3.0)
f. ≥ 2.5	Correct classification at week 12	Week 12	69.7	68.2	2.2 (1.2; 4.1)	77.8	52.9	1.7 (1.2; 2.3)
g. ≥ 3	ROC at week 12	Week 4	41.6	95.8	10.0 (1.4; 69.2)	57.1	77.5	2.5 (1.5; 4.3)
g. ≥ 3	ROC at week 12	Week 12	63.2	90.9	6.9 (1.8; 26.3)	71.1	64.7	2.0 (1.3; 3.1)
**RELATIVE CHANGE**								
a. ≥ 6%	75^th ^percentile at week 4	Week 4	88.3	54.2	1.9 (1.2; 3.0)	100.0	31.0	1.4 (1.2; 1.7)
a. ≥ 6%	75^th ^percentile at week 4	Week 12	96.1	45.5	1.6 (1.2; 2.6)	93.3	19.6	1.2 (1.0; 1.4)
b. ≥ 17%	ROC at week 4	Week 4	80.5	75.0	3.2 (1.6; 6.5)	100.0	46.5	1.9 (1.5; 2.3)
b. ≥ 17%	ROC at week 4	Week 12	94.7	59.1	2.3 (1.4; 3.8)	91.1	25.5	1.2 (1.0; 1.5)
c. ≥ 20%	Empirical	Week 4	76.6	75.0	3.1 (1.5; 6.2)	96.4	49.3	1.9 (1.5; 2.4)
c. ≥ 20%	Empirical	Week 12	90.8	59.1	2.2 (1.3; 3.7)	88.9	27.5	1.2 (1.0; 1.5)
d. ≥ 25%	Correct classification at week 4/75^th ^percentile at week 12	Week 4	76.6	75.0	3.1 (1.5; 6.2)	96.4	49.3	1.9 (1.5; 2.4)
d. ≥ 25%	Correct classification at week 4/75^th ^percentile at with 12	Week 12	88.2	63.6	2.4 (1.4; 4.2)	88.9	33.3	1.3 (1.1; 1.7)
e. ≥ 30%	75^th ^percentile at week 12	Week 4	67.5	87.5	5.4 (1.9; 15.7)	92.9	62.0	2.4 (1.8; 3.3)
e. ≥ 30%	75^th ^percentile at week 12	Week 12	84.2	63.6	2.3 (1.3; 4.1)	86.7	37.3	1.4 (1.1; 1.8)
f. ≥ 35%	ROC at week 12	Week 4	63.6	91.7	7.6 (2.0; 29.1)	85.7	64.8	2.4 (1.7; 3..5)
f. ≥ 35%	ROC at week 12	Week 12	81.6	72.7	3.0 (1.5; 6.0)	86.7	43.1	1.5 (1.2; 2.0)
g. ≥ 42%	Correct classification at week 12	Week 4	59.7	95.8	14.3 (2.1; 98.5)	82.1	69.0	2.7 (1.8; 3.9)
g. ≥ 42%	Correct classification at week 12	Week 12	72.4	72.7	2.7 (1.3; 5.3)	82.2	52.9	1.7 (1.3; 2.4)
h. ≥ 50%	Empirical	Week 4	53.2	100.0	ND	78.6	76.1	3.3 (2.1; 5.2)
h. ≥ 50%	Empirical	Week 12	67.1	86.4	4.9 (1.7; 14.3)	75.6	60.8	1.9 (1.3; 2.8)

^a^The values are those resulting from analysis of the data according to a specific methodology (for example, empiric smallest detectable difference, correct classification probabilities, 75^th ^percentile, ROC curve). ^b^Methodological technique used in the analysis to propose a potential threshold and (in the case of the ROC, 75^th ^percentile and correct classification probabilities) the time point during the study (for example, 4 or 12 weeks after initiation of etanercept therapy) such evaluation has been performed. ^c^The visit during the study (for example, either 4 or 12 weeks after initiation of etanercept therapy) during which the data collected were used to evaluate the external validity of the proposed thresholds. ^d^Patient's perspective based on the PASS question (X, Y and Z patients answered 'yes' at baseline, week 4 and week 12 respectively: Se, % patients with an absolute RAID score below the proposed cutoff who considered their condition to be acceptable (answering 'yes' to the patient acceptable symptom state (PASS) question) among all patients considering their condition to be acceptable; Spe, % patients with an absolute RAID score above the proposed cutoff who considered their condition to be unacceptable (answering' no' to the PASS question) among all patients considering their condition to be unacceptable. ^e^Physician's perspective based on the low disease activity defined by the disease activity score-erythrocyte sedimentation rate (DAS-ESR) < 3 (X, Y, and Z patients had a DAS < 3.2 at baseline, week 4 and week 12 respectively): Se, % patients with an absolute RAID score below the proposed cutoff and in low disease activity (for example, DAS28-ESR ≤ 3.2) at week 12 among all patients with low disease activity at week 12: Spe, % patients with an absolute RAID score above the proposed cutoff and with at least moderately active disease (for example, DAS28-ESR ≥ 3.2) at week 12 among all patients with at least moderately active disease at week 12. ROC, receiver operating characteristic; LR+, positive likelihood ratio.

**Figure 3:**
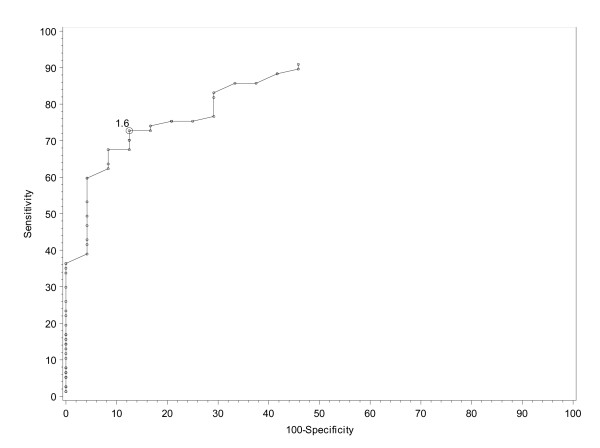
**Receiver operating characteristic curve showing absolute change in rheumatoid arthritis impact of disease (RAID) score at week 4**.

Figure 3 shows the ROC curves for absolute changes in RAID score after 4 weeks of therapy, resulting in an optimal threshold of 1.6. Similar analyses were performed for the absolute changes after 12 weeks of therapy and for the relative changes after 4 and 12 weeks of therapy, resulting in potential thresholds of 3, 17% and 35% respectively (Table 2).

**Figure 4:**
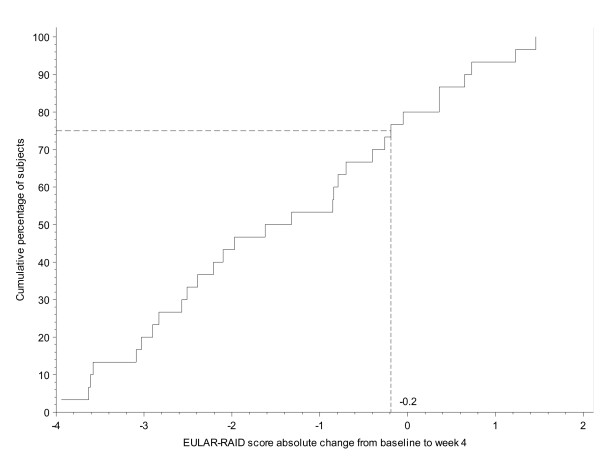
**Distribution of the absolute changes in rheumatoid arthritis impact of disease (RAID) score from baseline to week 4 in patients considering their condition to be slightly or moderately improved**. EULAR, European League Against Rheumatism.

**Figure 5:**
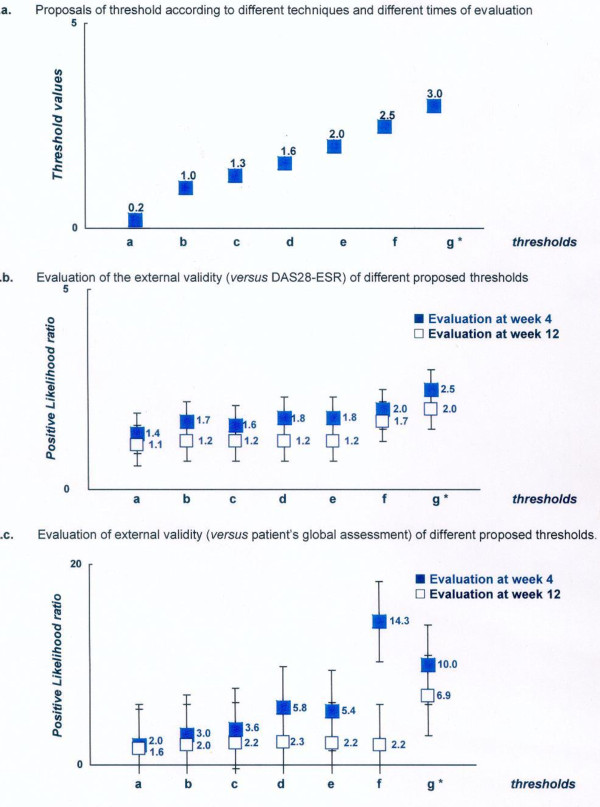
**Proposals and evaluation of different thresholds for defining a clinically meaningful improvement in an absolute change in the rheumatoid arthritis impact of disease (RAID) score**. **a**. Proposals of threshold according to different techniques and different times of evaluation. **b**. Evaluation of external validity (versus DAS28-ESR) of different proposed thresholds. **c**. Evaluation of external validity (versus patient's global assessment) of different proposed thresholds. *Thresholds, proposal based on the following techniques and time of evaluation: a, 75^th ^percentile technique at week 4; b, empirical technique and correct classification at week 4; c, smallest detectable change and 75^th ^percentile at week 12; d, ROC technique at week 4; e, empirical technique and correct classification at week 4; f, correct classification at week 12; g, ROC technique at week 12. ^+^Positive likelihood ratio (higher values are indicative of better performing thresholds. See Methods for further explanation). DAS28-ESR, Disease Activity Score 28-erythrocyte sedimentation rate; ROC, receiver operating characteristic.

Figure 4 plots the distribution of absolute change from baseline in RAID score after 4 weeks of etanercept therapy among the 30 patients considering their condition to be slightly or moderately improved. Using this technique, 75% of these patients had a RAID score below 0.2, and therefore 0.2 was proposed as a potential optimal threshold (Figure 5). Similar analyses were performed to evaluate the absolute changes after 12 weeks and also the relative changes after 4 and 12 weeks, resulting in potential thresholds of 1.3, 6% and 25% respectively (Table 2).

**Figure 6:**
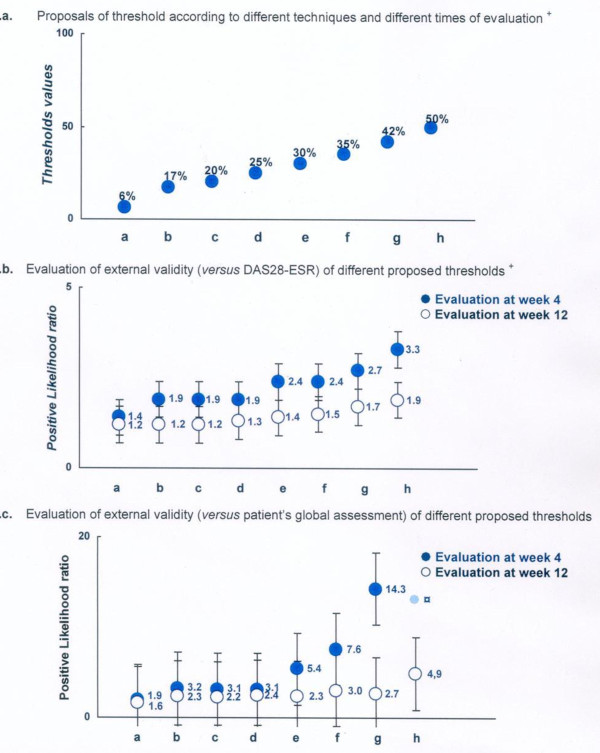
**Proposals and evaluation of different thresholds for defining a clinically meaningful improvement in a relative change in the rheumatoid arthritis impact of disease (RAID) score**. **a**. Proposals of threshold according to different techniques and different times of evaluation. **b**. Evaluation of external validity (versus DAS28-ESR) of different proposed thresholds. **c**. Evaluation of external validity (versus patient's global assessment) of different proposed thresholds. *Thresholds, proposal based on the following technique and time of calculation: a, 75^th ^percentile technique at week 4; b, ROC technique at week 4; c, empirical technique; d, correct classification at week 4 and 75^th ^percentile at week 12; e, 75^th ^percentile at week 12; f, ROC technique at week 12; g, correct classification at week 12; h, empirical technique. ^+^Positive likelihood ratio (higher values are indicative of better performing thresholds. See Methods for further information). DAS28-ESR, Disease Activity Score 28-erythrocyte sedimentation rate; ROC, receiver operating characteristic.

Table 2 and Figures 5 and 6 summarize the proposed thresholds resulting from these different techniques. These ranged from 0.2 (75^th ^percentile technique at week 4) to 3 (ROC technique at week 12) for defining a minimum clinically important improvement in the absolute change in RAID score and ranged from 6% (75^th ^percentile technique at week 4) to 50% (empirical technique) for a MCII in the relative changes in the RAID score.

#### Threshold for defining an acceptable status

**Figure 7:**
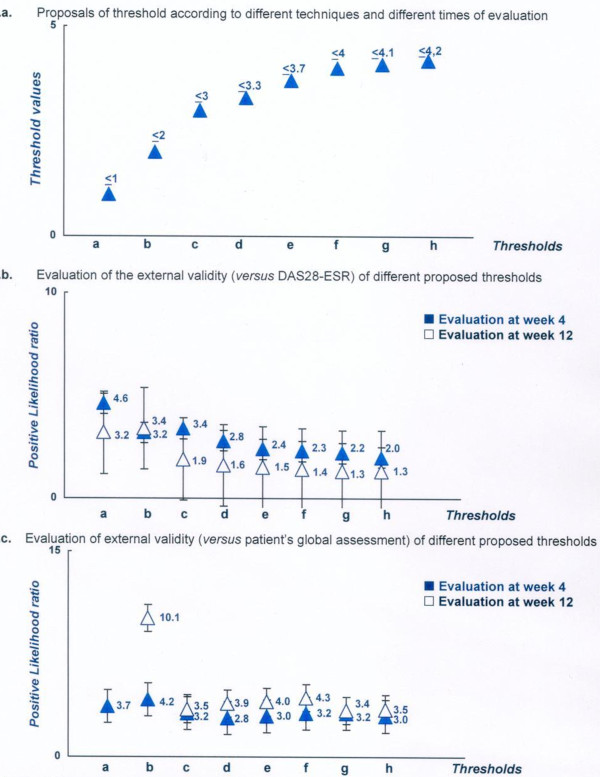
**Proposals and evaluation of different thresholds for defining an acceptable status according to the rheumatoid arthritis impact of disease (RAID) score**. **a**. Proposals of threshold according to different techniques and different times of evaluation. **b**. Evaluation of external validity (versus DAS28-ESR) of different proposed thresholds. **c**. Evaluation of external validity (versus patient's global assessment) of different proposed thresholds. *Thresholds, proposal based on the following technique and time of calculation: a, 75^th ^percentile technique at week 4; b, ROC technique at week 4; c, empirical technique; d, correct classification at week 4 and 75^th ^percentile at week 12; e, 75^th ^percentile at week 12; f, ROC technique at week 12; g, correct classification at week 12; h, empirical technique. ^+^Positive likelihood ratio (higher values are indicative of better performing thresholds. See Methods for further information). DAS28-ESR, Disease Activity Score 28-erythrocyte sedimentation rate; ROC, receiver operating characteristic.

Table 3 and Figure 7 summarize the thresholds proposed by the different techniques used, ranging from a minimal score of 1 (empirical technique) to 4.2 (75^th ^percentile and ROC technique at week 4) for the definition of a patient-acceptable symptom state in the RAID score.

**Table 3:** Elaboration and evaluation of the external validity of the different potential thresholds defining an acceptable status in the rheumatoid arthritis impact of disease (RAID) score

ELABORATION		Time of evaluation during the study^c^	EVALUATION					
Proposed threshold^a^			Patient's perspective^d^			Physician's perspective^e^		
			Se	Spe	LR+ (95% CI)	Se	Spe	LR+ (95% CI)
a. ≤ 1	Empirical	Week 4	21.2	94.3	3.7 (0.9; 15.4)	32.1	93.0	4.6 (1.7; 12.4)
a. ≤ 1	Empirical	Week 12	25.3	100.0	ND	31.1	90.2	3.2 (1.2; 8.1)
b. ≤ 2	Empirical	Week 4	36.4	91.4	4.2 (1.4; 13.1)	50.0	84.5	3.2 (1.7; 6.2)
b. ≤ 2	Empirical	Week 12	50.6	95.0	10.1 (1.5; 69.3)	66.7	80.4	3.4 (1.9; 6.2)
c. ≤ 3	Empirical	Week 4	54.5	82.9	3.2 (1.5; 6.8)	82.1	76.1	3.4 (2.2; 5.4)
c. ≤ 3	Empirical	Week 12	69.6	80.0	3.5 (1.4; 8.5)	80.0	56.9	1.9 (1.3; 2.6)
d. ≤ 3.3	75^th ^percentile at week 12	Week 4	63.6	77.1	2.8 (1.5; 5.3)	89.3	67.6	2.8 (1.9; 3.9)
d. ≤ 3.3	75^th ^percentile at week 12	Week 12	77.2	80.0	3.9 (1.6; 9.4)	82.2	47.1	1.6 (1.2; 2.1]
e. ≤ 3.7	Correct classification at week 12	Week 4	69.7	77.1	3.0 (1.6; 5.7)	92.9	62.0	2.4 (1.8; 3.3)
e. ≤ 3.7	Correct classification at week 12	Week 12	79.7	80.0	4.0 (1.6; 9.6)	84.4	45.1	1.5 (1.2; 2.0)
f. ≤ 4	ROC week 12	Week 4	72.7	77.1	3.2 ([1.7; 6.0)	92.9	59.2	2.3 (1.7; 3.1)
f. ≤ 4	ROC week 12	Week 12	86.1	80.0	4.3 (1.8; 10.4)	86.7	37.3	1.4 (1.1; 1.8)
g. ≤ 4.1	Correct classification week 4	Week 4	74.2	77.1	3.2 (1.7; 6.1)	92.9	57.7	2.2 (1.6; 2.9)
g. ≤ 4.1	Correct classification week 4	Week 12	86.1	75.0	3.4 (1.6; 7.4)	86;7	35.3	1.3 (1.1; 1.7)
h. ≤ 4.2	75^th ^percentile at week 4/ROC week 4	Week 4	77.3	74.3	3.0 (1.7; 5.4)	92.9	53.5	2.0 (1.5; 2.6)
h. ≤ 4.2	75^th ^percentile at week 4/ROC week 4	Week 12	87.3	75.0	3.5 (1.6; 7.5)	86.7	33.3	1.3 (1.0; 1.6)

^a^The values are those resulting from analysis of the data according to a specific methodology (for example, empiric smallest detectable difference, correct classification probabilities, 75^th ^percentile, ROC curve). ^b^Methodological technique used in the analysis to propose a potential threshold and (in the case of the ROC, 75^th ^percentile and correct classification probabilities) the time point during the study (for example, 4 or 12 weeks after initiation of etanercept therapy) such evaluation has been performed. ^c^The visit during the study (for example, either 4 or 12 weeks after initiation of etanercept therapy) during which the data collected were used to evaluate the external validity of the proposed thresholds. ^d^Patient's perspective based on the patient acceptable symptom state (PASS) question (X, Y and Z patients answered 'yes' at baseline, week 4 and week 12 respectively: Se, % patients with an absolute RAID score below the proposed cutoff who considered their condition to be acceptable (answering 'yes' to the PASS question) among all patients considering their condition to be acceptable; Spe, % patients with an absolute RAID score above the proposed cutoff who considered their condition to be unacceptable (answering 'no' to the PASS question) among all patients considering their condition to be unacceptable. ^e^Physician's perspective based on the low disease activity defined by the Disease Activity Score-erythrocyte sedimentation rate (DAS-ESR) < 3 (X, Y, and Z patients had a DAS < 3.2 at baseline, week 4 and week 12 respectively): Se, % patients with an absolute RAID score below the proposed cutoff and in low disease activity (for example, DAS28-ESR ≤ 3.2) at week 12 among all patients with low disease activity at week 12: Spe, % patients with an absolute RAID score above the proposed cutoff and with at least moderately active disease (for example, DAS28-ESR ≥ 3.2) at week 12 among all patients with at least moderately active disease at week 12. ROC, receiver operating characteristic; LR+, positive likelihood ratio.

### Evaluation of proposed thresholds

#### Evaluation of improvement thresholds

Table 2 summarizes the sensitivity, specificity and positive LR for each proposed threshold for the two external anchors (for example, patient's perspective and DAS28-ESR). These analyses showed that the positive LR was above 1 for all the proposed thresholds (Figure 5). However, the highest values were observed for a threshold of 3 for the absolute change (with a corresponding positive LR of 6.9 and 2.0 for the patient's perspective and DAS28-ESR external gold standards respectively, at week 12). Concerning the relative changes, the highest positive LR (4.9 and 1.9 for the patient's perspective and DAS28-ESR external gold standards respectively, at week 12) were observed for an improvement of at least 50% (Figure 6).

#### Evaluation of acceptable status thresholds

Table 3 summarizes the sensitivity, specificity and positive LR for each proposed threshold and for the 2 external anchors (for example, patient's perspective and DAS28-ESR). As for the improvement thresholds, all the thresholds proposed for defining an acceptable symptom-state had a corresponding positive LR > 1 (see Figure 7). For a maximum score of 2, the corresponding positive LR had the highest positive LR (for example, 10.1 and 3.4 for the patient's perspective and DAS28-ESR external gold standards respectively, at week 12).

## Discussion

This study clearly shows that the threshold value for a continuous variable, defining a relevant improvement or an acceptable symptom state, closely depends on the measurement technique. This first observation prompted us to perform a systematic validation of the proposed thresholds. In this study, we evaluated the validity of each proposed threshold by calculating the probability of being considered in good condition, by using external gold standards for the group of patients that were below or above the proposed threshold. We used two external gold standards reflecting both the patient's perspective (the patient's global assessment) and the physician's perspective (the DAS28-ESR) and calculated the positive LR: the best threshold was considered to be that with the highest observed positive LR. Using this methodology, we were able to propose an absolute change of at least 3, a relative change of at least 50%, and a maximum score of 2, as optimal thresholds for the RAID score, to define an absolute and relative MCII, and an acceptable symptom-state respectively.

This study has some weaknesses, but also several strengths. The very wide range of threshold values proposed using different methodologies raises the question of the optimal way to address this issue. All the techniques used in this study have been previously adopted, though no consensus has been reached in the field of clinical epidemiology [10-21]. This can be easily explained by the different rationales of each technique: the empirical technique involves asking physicians to propose relevant thresholds based on the simplicity of their proposal or their experience [20]. The aim of another technique is to avoid proposing a value below the measurement error of the outcome measure, as any interpretation of results using a threshold below the noise due to this measurement error is hazardous [22,23]. Finally, the techniques using an external anchor are also very relevant [14,15]. Although the validity of this external anchor may be questioned (here we used the previously reported gold standard MCII and PASS questions, which might raise the issue of a circular reasoning), these techniques make it possible to select the optimal threshold based on the arguments for and against, favoring sensitivity (for example, 75^th ^percentile technique [14,15]), sensitivity and specificity (for example, ROC curve and correct probability technique [23,24]). In this study therefore, we decided to use all the different techniques in a uniform group of patients (for example, active definite RA requiring a TNF blocker) receiving the same TNF blocker (etanercept). Despite this fact, we observed a very wide variability in the thresholds proposed by these analyses. From our point of view, such variability justifies a systematic evaluation of the validity of any proposed threshold and the main question is to define the optimal methodology for evaluating such validity. In this study, we approached this question by calculating the capacity of a proposed threshold to adequately classify a patient by considering previously validated external anchors from both a patient's perspective and a physician's perspective. The MCII and PASS questions were considered to be a gold standard anchor for the patient's perspective [14,15]. Because we also used the MCII and PASS questions for the elaboration of such thresholds, one might be concerned by the potential circular reasoning of this approach. This is why we decided to use not only this external anchor but also another one (the DAS28), which is considered a physician's perspective [26], while evaluating a patient. We then calculated the positive LR. This approach, using two different external anchors resulted in quite good concordance between the two analyses for each proposed threshold, strengthening our finding. This agrees with the results of a previous study suggesting that the PASS corresponds to moderate disease activity [29]. The data presented in the figures suggest also that the most stringent thresholds are also the most valid, at least with regard to our definition of external validity.

A weakness of this study was the fact that we were unable to evaluate the discriminant capacity of the proposed thresholds in order to validate them. Another potential weakness is that the proposed thresholds were defined in a single study with a relatively small sample size. On the other hand, the strength of this study is that all these different analyses of the definition of thresholds for a continuous variable were performed on a uniform group of patients. Despite these points, using our methodology and calculating the positive LR using two external anchors, we found a difference between the different thresholds, so that we were able to propose an absolute change of 3 points and a relative change of 50% for defining a clinically relevant improvement, and a maximum score of 2 for defining an acceptable status. Further studies in different patient populations, evaluating different facets of validity (including for example, the evaluation of discriminatory capacity), are necessary to confirm these proposals.

## Abbreviations

ACPA: anti-citrullinated protein antibody; CI: confidence interval; CRP: C-reactive protein; DAS: Disease Activity Score; ESR: erythrocyte sedimentation rate; EULAR: European League Against Rheumatism; ICC: intra-class coefficient of correlation; LR: likelihood ratio; MCII: Minimum Clinical Important Improvement; mHAQ: modified Health Assessment Questionnaire; OMERACT: Outcome Measures in Rheumatology; PASS: Patient Acceptable Symptom State; RA: rheumatoid arthritis; RAID: rheumatoid arthritis impact of disease; ROC: receiver operating characteristic; SDC: smallest detectable change; SDD: smallest detectable difference; TNF: tumor necrosis factor.

## Competing interests

This study was sponsored by Wyeth, which was acquired by Pfizer Inc (PARIS Cedex 14.FRANCE).

## Authors' contributions

MD participated in the protocol design, statistical analysis plan, data interpretation, and writing the manuscript. LG, DvdH, and TK all contributed to the statistical analysis plan and interpretation of data. YB and IL participated in the protocol design, statistical analysis plan, and data interpretation. All authors were involved in finalising the manuscript.
